# Terrestrial isopods associated with shallow underground of forested scree slopes in the Western Carpathians (Slovakia)

**DOI:** 10.3897/zookeys.801.24113

**Published:** 2018-12-03

**Authors:** Ján udy, Michal Rendoš, Peter Ľuptáčik, Andrej Mock

**Affiliations:** 1 Pavol Jozef Šafárik University, Faculty of Science, Institute of Biology and Ecology, Šrobárova 2, SK – 041 54 Košice, Slovakia Pavol Jozef Šafárik University Košice Slovakia; 2 Department of Ecology, Faculty of Humanities and Natural Sciences, University of Prešov, 17. novembra 1, SK – 081 16 Prešov, Slovakia University of Prešov Prešov Slovakia

**Keywords:** depth distribution, MSS, Oniscidea, shallow subterranean habitat, species diversity, subterranean traps

## Abstract

The shallow underground of forested scree slopes represents a little-studied subterranean biotope. In this paper, species diversity and depth distribution of terrestrial isopod communities studied in the depth profile (5–95 cm from the surface) of eight forested scree slopes in the Western Carpathians (Slovakia) is discussed. The southern edge of the Western Carpathians where the study sites are located represents the northernmost limit of distribution of obligate subterranean fauna in Europe. The sites differ from each other in type of bedrock, forest composition, slope aspect, and altitude. To sample isopods, a set of three subterranean pitfall traps consisting of a plastic cylinder (Ø 110 mm) and ten plastic cups was buried in each studied scree slope. In total, 252 isopods belonging to eleven species were sampled (1–5 species per site). Of the species found, the blind and depigmented *Mesoniscusgraniger* was the sole species closely associated with deeper parts of the depth profile and was present in most of the sites studied. Another ten species were represented by a small number of individuals and their occurrence deeper in the scree slope profile was rather accidental. A comparison between winter and summer periods indicates apparent differences in seasonal activities of isopods. Ethylene glycol seems to be more appropriate fixative solution for trapping isopods than formaldehyde.

## Introduction

An extensive labyrinth of air-filled dark voids among the rocky fragments found inside the forested scree slopes represents a peculiar type of shallow subterranean habitat (Culver and Pipan 2014). The mesovoid shallow substratum, as this habitat is ordinarily referred to, lies immediately below the soil, ranges from a depth of several centimeters up to several dozen meters and in the karst areas, it is interconnected with caves and narrow cracks situated deep below the ground surface (Juberthie et al. 1980, Giachino and Vailati 2010, Mammola et al. 2016). The soil and the forest growing above ameliorate fluctuations in temperature and humidity throughout the depth profile of scree slope. Leaves falling from trees onto the scree slope surface during autumn represent a rich and easily accessible source of nutrients that are brought to the scree slope interior either passively by percolating rainwater or actively by the migration of soil macrofauna (Gers 1998, Pipan et al. 2010). Due to environmental conditions that are intermediate between stable caves and variable surface, the mesovoid shallow substratum is populated, beside common soil dwelling species of invertebrates, by rare subterranean species, most of which possess morphological adaptations towards life in constant darkness such as anophthalmia, depigmentation, and elongation of appendages (Sket 2008, Nitzu et al. 2014, Jiménez-Valverde et al. 2015).

Communities of terrestrial isopods dwelling shallowly underground in forested scree slopes have been explored minutely so far only in the Czech Republic (Tuf et al. 2008), Romania (Nitzu et al. 2010, 2011) and Spain (Jiménez-Valverde et al. 2015). Comparing the species richness of mesovoid shallow substratum with the adjacent subterranean habitats, these studies found that the number of isopod species tend to decline in the gradient between soil and cave and some isopod species, particularly troglophiles, show high affinity to mesovoid shallow substratum.

Previous research in subterranean biology in the Western Carpathians has favored caves (Košel 2012, Kováč et al. 2014). Mock et al. (2015) and Rendoš et al. (2012, 2016a) conducted the initial thorough survey focused on diversity and depth distribution of subterranean invertebrate communities, including terrestrial isopods, at three model sites. In this paper, we summarize existing knowledge, both published and unpublished, of terrestrial isopod communities sampled intensely over the past years in the forested scree slopes along the southern edge of the Western Carpathians. Our aim was to (1) evaluate α and γ diversity of terrestrial isopod communities inhabiting interior of forested scree slopes, and (2) to describe the depth distribution of particular species with a special emphasis on subterranean species. Some methodological aspects are also discussed.

## Materials and methods

### Study sites

**Figure 1.**
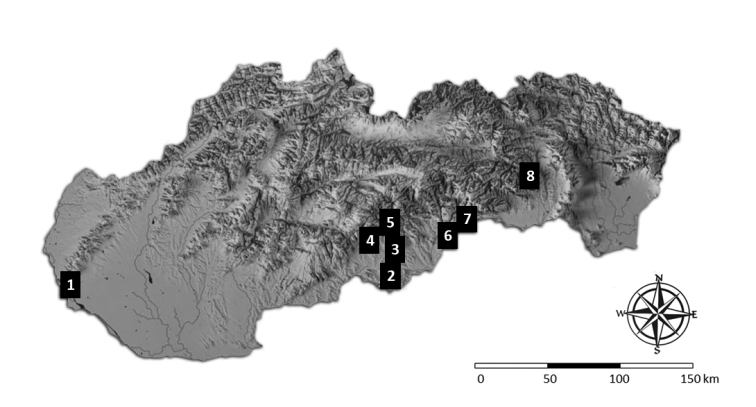
Location of the study sites. 1/ Borinka (Malé Karpaty Mts.), 2/ Belinské skaly (Cerová vrchovina Highlands), 3/ Drienok Valley, 4/ Slope next to the Malá drienčanská Cave, 5/ Collapse above the Veľká drienčanská Cave (three sites are in the Revúcka vrchovina Highlands), 6/ Vysoký vrch Hill, 7/ Doline next to Silická ľadnica Ice Cave (both sites in Slovak Karst), 8/ Malý Ružinok Valley (Čierna hora Mts.).

Our study was carried out successively from November 2008 to January 2016 on 8 forested scree slopes situated in 5 geomorphological units of the Western Carpathians (Slovakia). The studied scree slopes were predominately formed during the Pleistocene to Holocene by frost weathering and they differ in the type of bedrock (Figure 1 and Table 1), forest composition, structure of depth profile.

**Table 1.** Characteristics of scree slope sites studied. pH_(H20)_ and C_(ox)_ values, both after Rendoš et al. (2016b), were measured at four depths (5, 35, 55, and 95 cm). The numbers indicating particular studied scree slopes are stated in Figure 1.

**Locality**	**Bedrock**	**GPS coordinates**	**Altitude (m)**	**Exposition**	**Sampling period**	**Sampling days**	**pH_(H2O)_**	**C_(ox)_**
							**(5–95 cm)**	**(5–95 cm)**
**1**	Granitoid	49°77'N, 17°66'E	410	southwest	15 Jan 2015-16 Jan 2016	365	4.1-4.7	10.8-1.1
**2**	Basalt	48°13'N, 19°52'E	460	southwest	15 May 2012–17 Oct 2013	520	5-6.4	3.2-0.5
**3**	Limestone	48°32'N, 20°07'E	315	north	15 May 2012–17 Oct 2013	520	6.6-8.3	7.3-1.7
**4**	Limestone	48°29'N, 20°04'E	248	southwest	18 Oct 2013–4 Jun 2014	229	–	–
**5**	Limestone	48°29'N, 20°03'E	246	southwest	18 Oct 2013–4 Jun 2014	251	–	–
**6**	Limestone	48°31'N, 20°25'E	328	southwest	11 Jun 2014–29 Apr 2015	322	7.3-8.3	12.5-2.3
**7**	Limestone	48°33'N, 20°30'E	489	west	11 Jun 2014–29 Apr 2015	322	6.8-7.9	10-3.7
**8**	Limestone	48°50'N, 21°06'E	530	northeast	15 Nov 2008–15 Jul 2010	607	7.7-8.3	15.5-8.8

1 Scree slope in the Strmina Natural Reserve (Malé Karpaty Mountains) overgrown with beech forest (*Fagus*). This study site lies on acidic granitoid bedrock but in its immediate vicinity, there is a karst area (Borinka Karst) characterized by several karst formations such as caves and sinkholes. The scree slope profile has four distinct layers: litter and humus (0–5 cm), organo-mineral layer (5–20 cm), a layer consisting of mixture of mineralized soil and rocks (20–75 cm) and scree partially clogged with soil (75–110 cm).

2 Scree slope on basalt bedrock in the Belinské skaly National Nature Monument belonging to the Cerová vrchovina Highlands, an area known for the presence of a number of shallow pseudokarst caves. The scree slope profile is covered with a xerophilous oak-hornbeam forest (*Querco-Carpinetum*) and its profile consists of three different layers: litter and humus (0–5cm), organo-mineral layer (5–30 cm) and scree with spaces filled substantially with mineralized soils (30–110).

3 Limestone scree slope in the Drienok Valley (Revúcka vrchovina Highlands) situated a few meters below the entrance to the Špaňopoľská Cave. The site is surrounded by beech-hornbeam forest (*Fago-Carpinetum*) and in the scree slope profile, there are four distinct layers: leaf litter and humus (0–5 cm), organo-mineral layer (5–25 cm), mixture of rocks and soil (25–70 cm) and scree partly filled with soil (70–110 cm).

4 Limestone scree directly at the entrance to the Malá drienčanská Cave without apparent stratification of the profile. The interior of the scree includes a mixture of humus, soil and rocky fragments up to a depth of 30 cm. Below this, large compact boulders predominate.

5 Scree slope within the collapse above the Veľká drienčanská Cave. The slope profile without evident stratification consists predominately of rocky fragments originating from the previously collapsed cave ceiling. The spaces among the large limestone boulders are slightly filled with the mixture of soil and humus. Both sites (4 and 5) are located approximately 10 km west of the Drienok Valley (site 3) and are overgrown with beech hornbeam forest (*Fago-Carpinetum*).

6 Limestone scree slope in the Slovak Karst National Park situated about 30 m west of the entrance to the Ardovská Cave. The slope is overgrown with dogwood-maple forest (*Corno-Carpinetum*). On the scree slope surface, there are several large boulders, most of them covered by bryophytes. Three clearly separated layers are recognizable inside the scree slope: a layer consisting of litter and humus (0–15 cm) is followed by organo-mineral layer with admixtures of tiny rocks (15–75 cm) and scree formed by large rock fragments (75–110 cm).

7 Limestone scree slope along the doline near the entrance to the ice cave Silická ľadnica situated in the Slovak Karst National Park. The site is forested by linden-hornbeam, and maple (*Tilio-Aceretum* with *Carpinusbetulus*) trees and the scree slope profile is divided into three layers: leaf litter and humus (0–5 cm), thick organo-mineral layer (5–30 cm) and scree (30–110 cm).

8 Limestone scree slope in the Sivec National Reserve (Čierna Hora Mountains) including a massive limestone cliff with several shallow caves. The vertical profile of the scree slope covered with linden-maple forest (*Tilio-Aceretum*) is composed of the following layers: leaf litter and humus (0–15cm), organo-mineral layer (15–45 cm) clearly separated from scree (45–110).

### Sampling

To sample isopods, non-baited subterranean pitfall traps designed by Schlick-Steiner and Steiner (2000) and subsequently modified by Rendoš et al. (2016a) were used. Each trap consists of a plastic cylinder (length 110 cm, diameter 10.5 cm) circumferentially perforated with openings (Ø0.8 cm) in ten regular horizontal levels (5, 15, 25...95 cm). A removable set of ten plastic cups (volume 500 ml) filled with 4% formaldehyde or 50% ethylene glycol fixative solution is inserted into the plastic cylinder interior. The cups are placed right under the openings on the cylinder surface, enabling animals to be trapped at that particular level. At each studied scree slope, a triad of subterranean pitfall traps were placed 50 cm apart in a previously excavated pit over a meter deep. On the scree slope next to the Malá drienčanská Cave, and on the collapse above the Veľká drienčanská Cave (sites 4 and 5), we buried shorter (30 cm long) subterranean pitfall traps due to the presence of large boulders that prevented us from digging a deeper pit. Afterwards, the pit was backfilled to the maximum possible extent with the dugout soil and rocks in the original order of the layers and the cylinders were tightly closed by a plastic lid and covered by rocks and leaves found around the study site. To empty the traps and retrieve the sampled specimens, the set of plastic cups was pulled out of the cylinder; the content of each cup was poured into a plastic bottle and transported to the laboratory of soil biology (Institute of Biology and Ecology, PJ Šafárik University in Košice). The isopod material was later fixed in 75% ethyl alcohol and determined to the species level using several determination keys, such as Frankenberger (1959) and Radu (1985). Systematics and nomenclature of Isopoda species found follow the World Catalogue of Terrestrial Isopods by Schmalfuss (2003). The subterranean pitfall traps were emptied for the first time approximately a month after burial. This is, according to our previous experience, the period needed for regeneration of excavated soil layers and revival the locomotor activity of some sensitive species (Mock et al. 2015). Thereafter, the isopod sampling was timed so as to include at least one “cold” (winter) and one “warm” (summer) period of the year. The cold period refers to the months between October and April / May while warm period refers to the months between May and October. Sampling periods for each scree slope site studied are stated precisely in Table 1. In total, 195 traps were used.

### Community characteristics

To describe quantitative and qualitative characteristics of isopod communities, we calculated dominance, constancy, Shannon’s diversity index, and Pielou’s evenness index. The last two indices were first calculated for the material from each scree slope site separately and then collectively for the material from all sites. Due to the low number of isopods sampled, we were not able to perform more complex statistical analysis of our results. Dominance (D) was calculated by the formula D = 100*n/N where *n* is the number of individuals belonging to the specific species and *N* is the number of all individuals sampled. Constancy (C) was calculated using the formula C=100*pA/P where *pA* is the number of study sites on which, the specific species were sampled and *P* is the total number of study sites. Then after calculations, species were subdivided into the categories reflecting their dominance: subrecedent (D < 1%), recedent (D = 1–2%), subdominant (D = 2–5%), dominant (D = 5–10%), eudominant (D > 10%) and constancy: rare (C < 25%), widespread (C = 25–50%), constant (C = 50–75%), common (C > 75%). Shannon’s diversity index (*H*’) was calculated by the formula H = -∑(P_i_*lnP_i_), where *P_i_* is the fraction of the entire population made up of species *i* (proportion of a species is relative to a total number of species present, not encountered). Pielou’s evenness index (*J*’) was computed by the formula *J*’ = H’/H’_max_ = H’/lnS where *S* is the number of species encountered (Heip et al. 1998).

## Results

In total, 252 isopod specimens belonging to eleven species and six families were sampled from eight investigated scree slope sites (Table 2) The number of species sampled on individual study site varied from 1 to 5, with 2.8 species sampled on average per site. On the scree slopes near the Malá drienčanská and Veľká drienčanská caves, the values of Shannon’s diversity index equalled 0. The highest value of both Shannon diversity index and Pielou evenness index (*H*’=1.35 and *J*’=0.32) was recorded on the scree slope near the entrance to the Ardovská Cave. Among the captured Isopoda, *Mesoniscusgraniger* was characterized as common, because of its highest constancy (C = 75.0%, the species occurred at 6 of 8 sites); one species was classified as constant, three other species as widespread, and six species as rare. At the same time, *M.graniger* represented the only eudominant isopod species (D = 54.4%). The remaining species were classified as dominant (2 spp.), subdominant (3 spp.), subrecedent (3 spp.), and recedent (2 spp.) (Table 2).

**Table 2.** **List of sampled isopod species and community characteristics**. Abbreviations: C-Constancy: com-common, con-constant, wi-widespread, ra-rare; D-Dominance: ed-eudominant, do-dominant, sd-subdominant, re-recedent, sr-subrecedent. The numbers indicating particular studied scree slopes are stated in Figure 1.

Species	Study sites / ex									C		D	
	1	2	3	4	5	6	7	8	∑	(%)	degree	(%)	degree
*Hyloniscusriparius* (C Koch, 1838)	7	–	1	–	1	2	–	–	11	50	con	4.4	sd
*Lepidoniscusminutus* (C Koch, 1838)	–	–	–	–	–	25	–	2	27	25	wi	10.7	do
*Ligidiumgermanicum* Verhoeff, 1901	–	–	–	–	–	–	–	37	37	12.5	ra	14.7	do
*Ligidiumhypnorum* (Cuvier, 1792)	–	–	–	–	–	–	–	2	2	12.5	ra	0.8	sr
*Mesoniscusgraniger* (Frivaldsky, 1865)	–	7	15	40	–	16	14	45	137	75	com	54.4	ed
*Orthometoponplanum* (Budde-Lund, 1885)	–	–	–	–	–	4	–	–	4	12.5	ra	1.6	re
*Porcelliumcollicola* (Verhoeff, 1907)	–	–	–	–	–	17	–	–	17	12.5	ra	6.8	sd
*Porcelliumconspersum* (C Koch, 1841)	–	–	–	–	–	–	1	–	1	12.5	ra	0.4	sr
*Protracheoniscuspolitus* (C Koch, 1841)	–	2	1	–	–	–	1	–	4	37.5	wi	1.6	re
*Trachelipusratzeburgii* (Brandt, 1833)	2	–	–	–	–	–	–	–	2	12.3	ra	0.8	sr
*Trichoniscuscarpaticus* Tabacaru, 1974	–	–	1	–	–	–	–	9	10	25	wi	4.0	sd
∑ **ex.**	9	9	18	40	1	64	16	95	252	100	–	100	–
∑**spp.**	2	2	4	1	1	5	3	5	11	–	–	–	–
Shannon’s diversity index	0.53	0.53	0.63	0	0	1.35	0.46	1.11	–	–	–	–	–
Pielou’s evenness index	0.2	0.24	0.22	0	0	0.32	0.17	0.24	–	–	–	–	–

**Figure 2.**
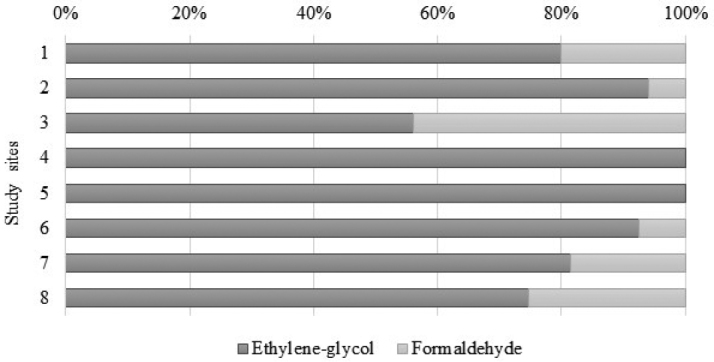
Ethylene glycol to formaldehyde ratio of sampled specimens from all study sites, where both fixating solutions were used.

Looking at depth distribution, the vast majority of species were exclusively sampled in the uppermost levels of the depth profile (5–15 cm). Only three species were distributed deeper in higher numbers, *M.graniger*, *Porcelliumcollicola*, and *Hyloniscusriparius*. The occurrence of other species in the lower parts of the scree slope profile was rather infrequent (Table 3). The individuals of *M.graniger* occurred abundantly in two parts of the depth profile: at the depths between 15–45 cm and 65–85 cm (Figure 2).

**Table 3.** A summary overview of the isopod depth distribution in the eight Western Carpathian scree slopes.

Depth (cm)	* H. riparius *	* M. graniger *	* L. minutus *	* Li. germanicum *	* Li. hypnorum *	* O. planum *	* P. collicola *	* P. conspersum *	* Pr. politus *	* Tra. ratzeburgii *	* T. carpaticus *	∑
5	2	1	26	36	2	4	7	–	2	2	10	92
15	3	14	–	1	–	–	4	–	2	–	–	24
25	–	25	–	–	–	–	1	–	–	–	–	26
35	–	29	–	–	–	–	1	1	–	–	–	31
45	–	18	–	–	–	–	1	–	–	–	–	19
55	3	7	1	–	–	–	–	–	–	–	–	11
65	–	11	–	–	–	–	–	–	–	–	–	11
75	1	11	–	–	–	–	1	–	–	–	–	13
85	–	14	–	–	–	–	1	–	–	–	–	15
95	2	7	–	–	–	–	1	–	–	–	–	10
∑	11	137	27	37	2	4	17	1	4	2	10	252

**Figure 3.**
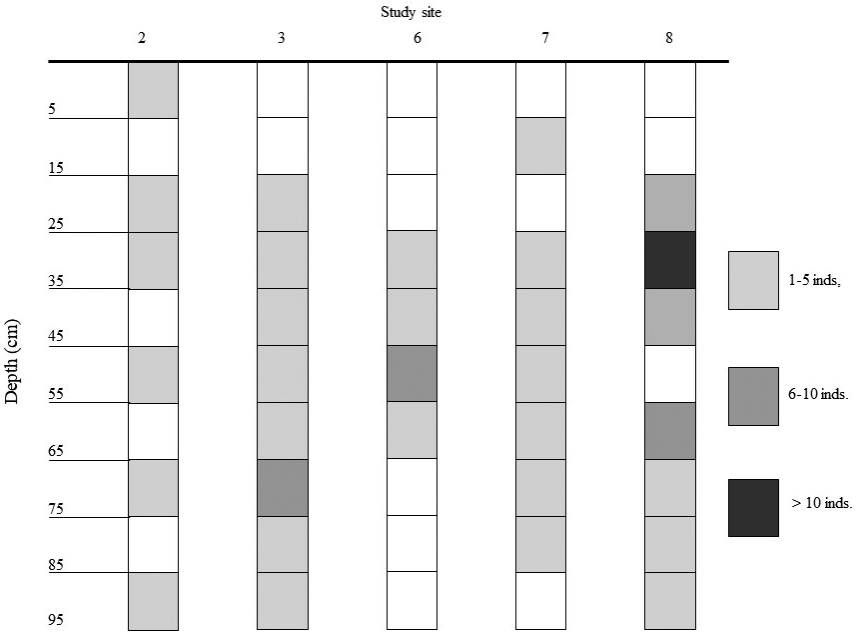
Depth distribution of *Mesoniscusgraniger*. Site 1 is out of the species range. Study sites 4 and 5 were not depicted, because whole depth gradient was not represented (see locality description).

**Figure 4.**
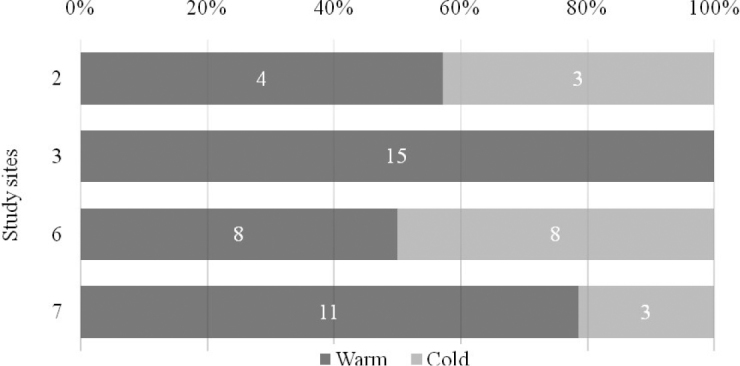
Comparison of warm and cold period sampling of *Mesoniscusgraniger* on study sites 2, 3, 6, 7.

Assessment of isopod seasonal activity was based on *M.graniger* sampled on two pairs of sites with the same duration of samplings: the first pair is represented by the scree slopes in the Belinské skaly and Drienok Valley (sites 2 and 3, respectively) while the second pair by the scree slopes next to the Ardovská Cave and ice cave Silická ľadnica (sites 6 and 7, respectively). On the sites 2 and 6, warm/cold (= May-October/November-April) season ratio was almost 50:50 but on the sites 3 and 7, markedly more specimens were collected during the warm sampling period. Taking into account all 4 study sites together, 70% of specimens were collected during the warmer sampling periods (Figure 3). Chi square test confirmed warm period to be a season of higher activity (Chi square test p-value 0.0009). As regards the comparison of effectiveness of two types of fixative solutions used in this study, ethylene glycol appears to be significantly more attractive (or less repellent) for isopods than formaldehyde (Figure 4).

Regarding pH, more study sites are located on limestone, thus soil pH is alkaline. Only two study sites have acidic soil pH. In comparison of abundance, on alkaline study sites 193 specimens were sampled, but on acidic sites only 18 (soil pH was not measured on study sites 3 and 4). Alkaline study sites are more diverse with average of four species on one study site, against two species on both acidic study sites. Total number of species on alkaline study sites is ten, on acidic study sites it is only four.

## Discussion

Rather scarce communities of terrestrial isopods, in terms of species number, were found to inhabit shallow depths on forested scree slopes in the Western Carpathians. This finding reflects the distributional pattern of terrestrial isopods in Europe with the most important hotspots situated in the Mediterranean regions (Manicastri and Argano 1989, Sfenthourakis and Taiti 2015). Eleven species documented within the studied forested screes represent approximately 20% of the entire Isopod fauna known from the territory of the W. Carpathians. All species can be considered as autochtonous and indicate the undisturbed state of the sites. They account for more than one third of all autochthonous isopod fauna in Slovakia (Frankenberger 1959, upgraded by our own unpublished data). Only two cavernicolous isopods are known from Slovakia: *Mesoniscusgraniger* (Mesoniscidae) and an undescribed blind species from the family Trichoniscidae (Kováč et al. 2014).

In the case of Isopoda, the typical number of species sampled in various surface habitats, such as forests, groves or pastures, ranges from 3 to7 (Farkas et al. 1999). The number of species sampled in the depth profile of forested scree slopes varied between 1 and 5 with average count of almost 3 species per study site. The lowest counts of species were recorded on the scree slopes built on basalt and quartzite rocks (two species). The most diverse isopod communities were observed on limestone bedrock (five species captured on a single site at the most). Two sites where shortened traps were buried revealed very low species diversity (one species captured per site). This was probably caused by the short and unsuitable period of subterranean pitfall traps exposition. A considerable similarity of the results was confirmed between the forested scree slopes and the caves from the same region. Some large-body forms of terrestrial isopods, frequently found in the entrance zone of caves, were missing in the screes (Papáč et al. 2009, Rendoš et al. 2016a). Differences in depth distribution of isopods among the studied scree slopes are ambiguous due to the low count of specimens sampled on the quartzite and basalt sites. What all studied scree slopes have in common is that the highest counts of specimens were sampled at depths of 5 and 15 cm. Associations of some isopods with basalt or quartzite bedrock is not assumed, since all species sampled on these two types of bedrocks were also found on the limestone scree slopes.

Relative abundance and depth distribution of isopods sampled inside the forested scree slopes clearly reflect the amount of organic residues along the depth profile, which tends to be the highest in the uppermost layers and to decrease downwards the depth profile as observed by Rendoš et al. (2016b). The vast majority of species were sampled in the nutrient-rich topsoil layers (5–15 cm) characterized by a high content of leaf litter and humus. Based on Sket’s (2008) classification of subterranean organisms, the isopods inhabiting these uppermost levels can be divided into two groups. The first group includes well pigmented trogloxenes: *Lepidoniscusminutus*, *Ligidiumgermanicum*, *Ligidiumhypnorum*, *Orthometoponplanum*, *Protracheoniscuspolitus*, *Trachelipusratzeburgii*, and *Trichoniscuscarpaticus* which penetrate cave and other subterranean habitats very occasionally. The smallest species, *T.carpaticus*, only recently documented in Slovakia (Rendoš et al. 2016a), is probably a hemiedaphic woodlouse. It does not penetrate any deeper underground in screes or caves in the Western Carpathians. It was described in Romania and is often present in caves and is considered to be troglophilous (Tabacaru and Giurginca 2013). The second group is represented by subtroglophiles *Hyloniscusriparius*, *Porcelliumcollicola*, and *Porcelliumconspersum* possessing no morphological adaptations to life in dark subterranean environment. These mostly surface dwelling species often use deeper soil horizons to overcome drought. Deeper parts of the scree slope profiles (from 15 cm downwards), characterized by a stable microclimate and much higher organic matter content than in deep caves, are almost exclusively inhabited by *Mesoniscusgraniger* – an eyeless and depigmented eutroglophile often found in subterranean habitats of the Carpathian and Dinaric Mountains (Mock et al. 2005, Giurginca 2009, Bedek et al. 2011, Tabacaru and Giurginca 2013)

As regards seasonal activity of *M.graniger*, our results prove the warm period of the year (vegetation period) to be the seasons with higher activity of *M.graniger*. Seasonal activity of other species was not assessed, due to their low abundance. This indirectly suggests that there is no massive seasonal vertical migration of the isopods into deeper layers of the forest scree slopes. Migration of terrestrial isopods into the deep soil is not necessary, since they can spend winter periods at shallow depths. Fallen leaves and snow cover provide a sufficient isolating layer to prevent lower lethal temperatures to isopods from being reached. Avoiding the uppermost layer of substrate, where the temperature has fallen below freezing point for several winter months, as overwintering isopod strategy could be minimized to move to a depth of a few centimeters under appropriate conditions, when the surface of soil is covered by fallen leaves and snow (Tanaka and Udagawa 1993).

Comparison of fixative solutions ended up more positively for ethylene glycol in which, more than 50% individuals of Isopoda were sampled. This was probably caused by the repelling effect of formaldehyde, since ethylene glycol was confirmed to show neither strong repellent nor strong attractive effect on arthropods (Gerlach et al. 2009). Another possible cause of lower number of Isopoda caught by formaldehyde traps could be also the non-mixing of detergent into formaldehyde solution. Using attractive bait in the traps, highly favored by coleopterists, will bring more faunistic knowledge, including unique findings (Mammola et al. 2016), but without the possibility to interpret the depth preferences of invertebrates.

It is not clear from our research, how pH is affecting terrestrial isopods assemblages, since our species and specimens counts are very low. If we take a look at the dominant *M.graniger*, we can see that this species is not affected by pH, due to the presence of this species on both alkaline and acidic study sites. The reason why this species is missing from study site 1 is that it is outside the distribution area of this species (Košel 2012). Terrestrial isopods need calcium, because of their exoskeleton structure (Zimmer et al. 2000). Limestone bedrock is a very good source of calcium ions, so this could be a reason why alkaline study sites (on limestone bedrock) are more abundant and diverse. Soil pH is not the sole reason of this phenomenon. We assume that it is a combination of soil pH, temperature, humidity and other externalities.

### Conclusions

The shallow underground of forested scree slopes in Slovakia is not inhabited by unique terrestrial isopods, but is uncommonly visited by surface (forest) species or by the sole subterranean species sampled (*Mesoniscusgraniger*). The interior of forested scree slope can be considered as a part of subterranean environment inhabited in the long term by fauna specialized to live in permanent darkness. Eleven species were sampled in total, which is more than one third of the autochthonous isopod fauna in Slovakia. Well-preserved status of scree habitats is supported by the presence of the rare species, *Mesoniscusgraniger*, *Orthometoponplanum*, and minute Carpathian endemic *Trichoniscuscarpaticus*. Subterranean pitfall traps with ethylene glycol proved themselves as suitable apparatuses to collect macrofauna from this environment.
